# Current state of the art, multimodality research and future visions for the treatment of patients with prostate cancer: consensus results from “Challenges and Chances in Prostate Cancer Research Meeting 2013”

**DOI:** 10.1186/s13014-014-0224-4

**Published:** 2014-11-04

**Authors:** Stephanie E Combs, Jürgen Debus, Günter Feick, Boris Hadaschik, Markus Hohenfellner, Roland Schüle, Jens-Peter Zacharias, Malte Schwardt

**Affiliations:** Technische Universität München (TUM), Klinikum rechts der Isar, Department of Radiation Oncology, Ismaninger Straße 2, 81675 München, Germany; Bundesverband Prostatakrebs Selbsthilfe e.V., Alte Straße 4, 30989 Gehrden 3, Germany; Department of Urology, University Hospital Heidelberg, Im Neuenheimer Feld 110, 69120 Heidelberg, Germany; Universitätsklinikum Freiburg, Urologie, Hugstetter Strasse 55, 79106 Freiburg, Germany; Department of Radiation Oncology, University Hospital of Heidelberg, Im Neuenheimer Feld 400, 69120 Heidelberg, Germany

## Abstract

A brainstorming and consensus meeting organized by the German Cancer Aid focused on modern treatment of prostate cancer and promising innovative techniques and research areas. Besides optimization of screening algorithms, molecular-based stratification and individually tailored treatment regimens will be the future of multimodal prostate cancer management. Effective interdisciplinary structures, including biobanking and data collection mechanisms are the basis for such developments.

## Introduction

Treatment of patients with prostate cancer has improved over the last decades, based on the understanding of tumor biology, molecular characteristics of tumors, improvement in surgical techniques as well as innovations in radiation oncology. Today, treatment of prostate cancer is an interdisciplinary task involving several treating disciplines and in some situations competing treatment options. Every modality is associated with distinct risk-benefit profiles, and intensive patient counseling as well as clinical and imaging findings are the basis for treatment decisions.

Decades ago treatment usually was limited to radical surgical resection of the prostate or simple conformal radiation therapy being associated with side effects, such as incontinence, bowel dysfunction and impotence, among others. Nowadays, improved anatomic nerve-sparing surgical approaches, development of robot-assisted surgery and intensity modulated radiation therapy have improved the risk-benefit ratio. For certain indications, large randomized studies have shown that adjuvant radiotherapy can increase progression-free and overall survival. In general, radiotherapy has become a valid treatment alternative in patients with prostate cancer, and with definitive treatments applied with high-end radiotherapy long-term curation can be achieved.

As individualized treatments are emerging, evaluation of molecular markers in tumor tissue, epigenetic factors or other risk constellations become more relevant and are focus of several research strategies. Biobanking structures are essential to expand knowledge in this regard. In the future, this novel information may help stratify patients for certain treatment modalities, treatment intensification or prevent subgroups of patients from over-treatment.

To bundle all these relevant data and innovative concepts, a consensus meeting in 2013 funded by the German Cancer Aid was held bringing together experts in the relevant fields for prostate cancer treatment and to lay the basis for future structured research concepts. The aim of the present manuscript is to summarize state-of-the art treatment, recent research results as well as prospective strategies for the future discussed in this framework. The manuscript should serve as a reference basis for interdisciplinary prostate cancer researchers in the fields of medicine, biology, physics as well as health care management.

## State of the Art Urology

Prostate cancer remains the most common malignancy and the second most common cause of cancer related death in men (Surveillance Epidemiology and End Results: SEER). Since the introduction of blood based PSA testing in the 1970’s the prostate cancer incidence significantly increased until the 1990’s but remained stable over the last ten years (SEER). State of the art therapies currently lead to 5- and 10-year survival rates of 100% and 98% respectively (SEER). The effect of PSA screening on the high survival rates is still discussed controversially between two leading studies in the United States (PLCO) and Europe (ERSPC). Both studies have enrolled large number of men (PLCO: 77.000/ERSPC:162.000) and have reported follow-up of 13 and 11 years respectively until today. While the PLCO study does not show any significant reduction in prostate cancer related mortality in the screening arm, the ERSPC study does show reduction in mortality of up to 29% [1]. Consensus on the data of ERSPC show clearly that beneficial effects from screening only become visible after a period of several years of follow up, as reported in the New England Journal of Medicine in 2012. Critics are especially on the PLCO study (high contamination of screened men outside the trial within the control arm), which limit the quality of the conclusions, which can be drawn from the study. But also the ERSPC study concludes despite the mortality reduction that more data especially on adverse effects of screening and cost effectiveness have to be acquired to make a final recommendation [1]. Several publications following the report of the two studies came to the same conclusion that individual decisions for screening have to be made between the patient and his physician [2-4]. This consensus opinion was strongly supported especially by the coordinators of the ERSPC study. In general, “the patient has to come first” in an individualized decision process for screening and treatment.

Despite the high 5- and 10 year survival rates prostate cancer mortality is estimated to be 29.000 cases with 238.000 newly diagnosed cases for 2013 (SEER). To identify and treat those patients, who have a tumor, which will cause severe symptoms or will be lethal is the persisting challenge in the daily routine of urologists and oncologists. At the same time unnecessary invasive diagnostic procedures or treatments have to be minimized [1].

Once a tumor has been diagnosed and validated in histological biopsies several treatment options especially for the organ-confined tumor are available. To prospectively compare the different regimens available, prospective multicenter trials such as the German PREFERE Study (NCT01717677) are required. In this four arm preference based study, surgery (radical prostatectomy), radiation (external beam or permanent seed implantation) and active surveillance will be compared in terms of effectiveness and side effects in a multicenter randomized trial including over 7000 men.

The surgical technique has been refined to the extent of robot assisted minimally invasive systems (DaVinci), which deliver 3D vision to the surgeon and level out even minimal shaking of his hands [5]. Open and minimally invasive nerve sparing operations are standard of care, if the tumor grade permits it [6]. Simultaneously, active surveillance has gained more attention due to increasing rates of overtreatment in the face of the large numbers of low risk tumors detected through PSA-testing. The ERSPC study estimated the percentage of over-diagnosed men in the age group 55–74 to be as high as 50% [1]. Active surveillance requires continuous follow-up to measure PSA kinetics and to re-evaluate tumor progression by invasive needle biopsies. Increasing Gleason scores, increase tumor volume and rapidly increasing PSA values are widely accepted trigger points for treatment. Controversy of PSA measurement and needle biopsy frequency is still ongoing, since follow-up time in active surveillance clinical trials is still too short. Moreover, the promising potential of multiparametric magnetic resonance imaging (MRI) of the prostate to non-invasively monitor tumors over time is gaining exponential attention.

Focus in the future has to be on additional diagnostic parameters to clearly identify low risk tumors, which will not need treatment and separate them from high risk tumors, which do need aggressive treatment. It is believed that the heterogeneous outcome of prostate tumors is based on different tumor subtypes, which could be defined by certain molecular properties. Currently, several national and international institutions have set up large efforts to further delineate the molecular heterogeneity of prostate tumors.

To “bridge the gap from mind to market” multidisciplinary efforts are needed that include clinicians as well as academia and industry. Molecular screening of human samples, target validation in in-vitro and in-vivo systems, protection of intellectual property and optimization of candidate compounds through biotech spin-offs for initiation of clinical trials within the same institution can lead to successful novel therapies. Among others, the clusterin inhibitor Custirsen (OGX-011) has been developed in such a way at the Vancouver Prostate Centre and is currently evaluated in phase III clinical trials.

Data collection at clinical high volume centers can lead to multiparameter assessment of clinical and molecular data. This provides a basis to successfully implement risk assessment scores to sub classify prostate tumors. Tissue microarray platforms including thousands of patients can be screened using various ‘-omics’ approaches to identify novel marker signatures for different tumor subtypes. Thereby surgical samples are being translated into clinically applicable diagnostic tools. An additional level of tumor alterations called ‘epigenetics’ is currently being explored in one of Germany’s largest collaborative research centers MEDEP (medical epigenetics) at Freiburg University Hospital. The collaboration follows a multidisciplinary approach to define epigenetic diagnostic and therapeutic tools for prostate cancer.

## State of the Art Radiation Oncology

Radiotherapy has been established as a clear treatment alternative in patients with prostate cancer. This is due to the significant developments over the last decades, continuously increasing precision of dose delivery to the tumor while sparing normal tissue and thus reducing treatment-related side effects.

The biggest step was the development of 3D-CT-based treatment planning, which is considered the treatment standard today. With this, dose conformality to the prostate and radiation doses could be increased. An early study evaluated this possible dose escalation in patients with prostate cancer, and could show that higher doses increase biological progression-free survival independently from tumor stage and risk stratification, while side effects where rather reduced compared to older treatment techniques [7]; this was confirmed also in long-term follow-up. The improved biochemical control of higher doses at 74–80 Gy compared to <70 Gy have been confirmed by several groups [8-11]. Subsequent improvements included further modulation of photon beams, such as Intensity Modulated Radiotherapy (IMRT; [12,13]). Here, each individually formed radiation field is in itself inhomogeneous, adapting the dose within the field configuration to anatomical requirements. IMRT significantly helped decrease toxicity when applying high-dose radiotherapy to the prostate; even with doses above 80 Gy only moderate side effect, e.g. to the rectum, are observed [11,14-16]. For intermediate-risk and high-risk patients, however, 5-year biochemical relapse free survival remains between 30-45%, thus further treatment optimization is required. Recently, further dose escalation trials beyond 80 Gy have been conducted, and even ultra-high doses of 86.4 Gy are being evaluated. Spratt et al., reported only recently long-term follow-up data after a median of 5.5 years, with 7-year biochemical relapse free survival of 98.8%, 85.6% and 67.9% for low- intermediate and high-risk patients [17]. However, for most approaches real long-term follow-up still remains to be awaited.

For further improvement of the therapeutic window, modern radiation machines are coupled with imaging, such as in-room CT-scanners, or radiation and imaging in one machine. Daily imaging prior to treatment is possible, correcting potential positioning errors of the patient. Currently, adaption of dose distribution not only to interfractional movement, but also to intra-fractional changes is possible. This means, dose distribution can follow movement and deformation of organs at risk (OAR), of the target volumes or target organs (Adaptive Radiotherapy, ART). Research is currently establishing technical advancements such as gating (radiation is on when the target is within a certain range of the movement amplitude) or tracking (radiation follows movement of the target) [13]. Combinations of radiation and imaging in terms of Image Guided Radiotherapy (IGRT) have lead to further increase of dose in prostate cancer treatment, thus further increasing outcome [12,18,19]. With these technical developments, also local dose increase to e.g. positive lymph nodes in patients with pelvic nodal disease can be targeted precisely, e.g. based on PSMA-PET-Imaging.

The dose-limiting OAR in the treatment of prostate cancer is the anterior rectal wall, lying directly posteriorly to the prostate. Thus steep dose gradients to the rectum are essential. One means to spare the rectum from dose is application of a sterile gel between the prostate and the rectum, providing several millimeters of space to produce a dose gradient [20]. Such gel applications together with highly advanced treatment techniques contribute beneficially to the risk to benefit profile.

Novel treatment modalities in radiotherapy may lead to further improvements. Particle therapy offers physical as well as biological benefits, the latter for high-LET (Linear Energy Transfer) particles. When entering the patient particles deposit very low energy, followed by steep dose deposition in the so called Bragg Peak. The depth of the Bragg Peak is energy-dependent. Thereafter a sharp-fall-off spares tissue behind the target volume additionally. Due to these physical properties the integral dose to the patient can be reduced, and the dose conformality can be enhanced. This is about comparable for protons and carbon ions, while carbon ions are high-LET particles associated with a higher relative biological effectiveness. Due to the biological properties of prostate cancer tissue with a high α/β value, there is substantial rationale for the use of the carbon beam. Additionally, increase of daily dose and reduction of overall treatment time (hypofractionation) is feasible, which from a radiobiological perspective seems to be favorable for prostate cancer tissue. To date, no randomized trial on the real value of particle therapy is available. One study in the USA has compared standard treatment to a dose-escalation using a proton boost in the experimental arm; this study has shown increase in outcome for the high-dose arm, however, such dose escalation is today potentially possible using IMRT/IGRT approaches not available at the time of initiation of that trial [11,14,16]. Thus, critics argue this study has only compared high-dose versus low-dose. However, the data remain a randomized trial comparing proton dose escalation, and further studies must still show that outcome and toxicity are comparable with photon dose escalation [16]. Future study designs must aim at comparison of these highly advanced techniques in patients with prostate cancer [21].

## Imaging and staging

While over years CT-based diagnostics as well as ultrasonography were considered the imaging standard in prostate cancer, multiparametric MRI as well as novel molecular imaging based on PET or SPECT imaging contribute significantly to differential staging, and enable individualized treatment strategies.

Currently, the basis for staging of high risk disease remains to be contrast-enhanced CT-imaging to describe the extent of nodal involvement in the pelvis, as well as to identify bony lesions. Staging should be completed by bone szintigraphy to rule out bone metastases. Emerging functional imaging, including diffusion-weighted MRI and dynamic contrast-enhanced MRI, MR-spectroscopy, lymph-node specific contract agents as well as PET-imaging using novel tracers can provide novel insights and potential for treatment improvement. Improved nodal characterization together with size and location of high-risk sub-regions or identification of prostate-cancer affected regions within the prostate provide great potential for the future.

Over recent years, PET-Imaging with ^11^C-choline has emerged as a promising diagnostic tool for patients with prostate cancer. It has been shown that ^11^C-choline has a high sensitivity in prostate cancer patients, and clinical evaluations have shown a better diagnostic accuracy for choline PET in comparison with other conventional imaging modalities; early detection of recurrences may lead early treatment and thus improved overall outcome. Lately, PET/CT and hybrid PET/MRI with Gallium 68 prostate-specific membrane antigen (PSMA) ligands have shown promising results with further improved sensitivity as compared to choline tracers [22,23] Based on such molecular imaging, not only early and accurate diagnoses become possible, but also targeted treatment can be implemented: endoradiotherapy and radiation therapy with integrated boost concept to PET-positive lesions for primary definitive treatment, or localized small-volume radiotherapy to PET-positive lesions are worth investigating and are currently evaluated within prospective clinical trials.

## Biobanking and epidemiology

In order to develop novel diagnostic or therapeutic targets their validation in large numbers of tissue samples is necessary. As already demonstrated in the previous topics, tissue derived data can be very rewarding but they require an enormous degree of planning, maintenance and funding resources in order to provide high quality biological material and data over a long period of time. Not only the sample collection, histologic validation, storage, inventarisation and the retrieving algorithm are important but also the connection to clinical data and its continuous updating [24]. An example of the power of large-scale tissue microarray (TMA) analysis was presented: a collaborative group a researchers from Heidelberg and Hamburg compared genomic alterations in 11 early onset tumors from younger patients to 7 late onset tumors from elderly patients and discovered a preference of androgen driven structural genomic alterations (e.g. TMPRSS-ERG fusion) in the early onset tumors. These initial findings were validated using TMA technique representing over 10.000 patients’ tissue samples and confirmed the original findings [25]. There was strong consensus that high quality tissue biobanking is of high interest and importance and should be seen as an investment in the future of improving disease diagnostics and treatment in the framework of all cancer centers.

Apart from novel targets on the molecular level classical family history also provides valuable information in risk assessment of prostate cancer: Fallah and colleagues incorporated the degree of relationship (first degree or second degree relative) and the co-occurrence of prostate cancer, breast cancer or esophageal cancer into a score (PCRAM: prostate cancer risk assessment model; [26]) to optimize the starting point for prostate cancer screening.

## Patient stratification and biomarkers

The introduction of large-scale next generation sequencing techniques into cancer research opened a whole new research field. The results however suggest that the heterogeneity among prostate tumors is far higher than anticipated before [27]. Analysis of single structural alterations might not be sufficient to characterize a whole tumor. Multilayer analysis of structural genomic alterations, mutations, gene-fusion events, methylation status and the resulting gene expression will have to be analyzed in individual patients to determine optimal risk adapted treatment. Several groups have followed research in this field: Rubin et al. [28] followed this approach recently and deep sequenced 57 prostate cancer genomes together with benign control tissue. They discovered that several structural genomic alterations were derived from a single event. This mechanism established a large number of structural alterations in a coordinated fashion in cancer tissue caused by only a few catastrophic events. They termed this mechanism chromoplexy. The model of chromoplexy will help to understand the complex genomic rearrangement found in prostate cancer. The use of next generation sequencing techniques can help in the future to stratify prostate cancer. Rubin stated, that despite the huge amount of sequencing data provided by international and national genome projects such as the International Cancer Genome Consortium (ICGC) or The Cancer Genome Atlas (TCGA) it is essential to filter out the meaningful alterations and put them into a context of cellular processes, which can be targeted [25].

The question, if disrupted signaling pathways may influence or even cause genomic instability is in focus for several research groups: in Heidelberg Duensing and colleagues have discovered a number of factors, which disrupt the integrity of mitosis leading to aneuploidy such as the fibroblast growth factor 2 (FGF2), centrosomal protein 57 (CEP57) and polo-like kinase 4 (PLK4) [29,30]. Duensing and his team hope to use theses molecules as novel biomarkers in early stages of cancer diagnostics.

Another approach follows the search for novel biomarkers however on the epigenetic level. The teams around Büttner and Schüle et al. found that the levels of lysine specific demethylase 1 (LSD1) predict the clinical behavior of prostate cancer [31]. Further unraveling of the mechanism of action of this enzyme and its co-factors might open novel diagnostic and therapeutic options. Rather than sequencing the whole genome epigenomic analysis sequences only those DNA sections, which are occupied by DNA modifying enzymes such as e.g. LSD1. None of the above projects would have been possible without the analytical power of bioinformatics. Manke and colleagues co-developed a computational tool to predict the DNA binding affinity for DNA binding molecules such as transcription factors to better understand and predict the presence of combinations of transcription factors at any given gene regulatory sequence [32].

Biostatistics is a key player in all tissue- and molecular-based analyses: novel programs and accessible tools to visualize multilayer data for scientific use, e.g. by circus plots, are currently under investigation. Large-scale computational analysis recently revealed that younger patients have smaller tumors but higher percentage of driver mutations compared to tumors in elderly patients [25]. The implications of genetic counseling based on such data will have to be discussed with great care in the future.

The vast technical possibilities opened up by next generation sequencing technologies, high volume tissue biobanking resources and computational analysis power offers promising novel pathways to pursue diagnostic and therapeutic strategies.

## Systemic treatment

For advanced disease, several options in terms of systemic treatment are available. This includes androgen deprivation therapy, chemotherapy, and novel molecular targets [33,34]. The oldest player in this context is certainly adrogen deprivation therapy: Continuous androgen deprivation is often continued indefinitely regardless of additional therapies, however at some point may not lead to a continuous tumor control. In addition, some therapies to be offered additionally have demonstrated survival and quality-of-life benefits: This includes abiraterone acetate and prednisone, enzalutamide, or Radium-223 in patients with bone metastases. For chemotherapeutic agents, docetaxel and prednisone are often administered. In this field several prospective clinical trials are under evaluation, especially with focus on the question when to offer androgen deprivation therapy (early or later during the disease course), and when to switch to other systemic options.

## Prospective trials

In the era of personalized medicine prospective clinical trials are required to identify the role of molecular markers, specific tumor and normal tissue characteristics or epigenetic factors on the course of prostate cancer. To achieve this, large multi-center collaborative structures including biospecimens should be planned and conducted. For these, intelligent trial design on smaller patient subgroups as well as large prospective clinical trials are required to compare existing treatment alternatives. The role of biologically stratified treatment decisions must be validated for individual patient cohorts.

Initiatives such as the German PREFERE study for low- and early intermediate-risk prostate cancer patients, as well as cohort studies and prospective registers on active surveillance and wait-and-see strategies further characterizing the natural course of prostate cancer are currently ongoing, and will provide scientific results in the near future. Lacking to date are real comparisons of surgery and radiation therapy, for intermediate risk or high-risk tumors. For such trials, not only commitment of the surgical and radiation community are necessary, but also patient willingness to undergo randomization procedures. Often patients have a strong personal preference and therefore feel uncomfortable undergoing randomization. However, extensive information about the treatment characteristics as well as the specific side effects, benefits or advantages of the different treatment alternatives may help develop such trial concepts. Within single disciplines, direct comparison of alternative modalities or concepts are also warranted: To date, no study has shown superiority robot-assisted surgery compared to open surgery performed by a highly experienced surgical team. In radiation oncology, long-term data of very high-dose radiation therapy coupled with IGRT are being awaited, and the most recent controversy about the value of particle therapy for the treatment of prostate cancer requires a direct comparative prospective trial of particle therapy with high-end photon treatment.

## Summary and conclusion

The two-day workshop funded and organized by the German Cancer Aid provided an informative platform for exchange of novel data and innovative treatment concepts. Current state-of-the-art treatment recommendations in the different disciplines were presented and discussed. A strong consensus was set on the future of individualized treatment, taking into account molecular signatures of tumor tissue for stratification. In the era of personalized medicine, each medical discipline should tailor their treatment to the individual needs and preferences of the single patient.
